# Integrating Lived Experience in Preprofessional Training in Speech Pathology and Audiology

**DOI:** 10.1111/tct.70183

**Published:** 2025-08-25

**Authors:** Rachel Glade, Saira Hussain, Claire Wilkes, Christine Hunter, Faheema Mahomed‐Asmail

**Affiliations:** ^1^ Department of Communication Disorders and Occupational Therapy University of Arkansas Fayetteville Arkansas USA; ^2^ Department of Audiology, College of Health and Life Sciences Aston University Birmingham UK; ^3^ Hearing Matters Australia Sydney Australia; ^4^ Department of Speech‐Language Pathology and Audiology University of Pretoria Pretoria South Africa

**Keywords:** audiology, clinical training, lived experiences, person‐centred care, pre‐graduates, reflective practices, speech pathology

## Abstract

**Background:**

Incorporating persons with lived experience (PWLE) has proven effective in supporting preprofessionals in their training by providing authentic insights that foster empathy, compassion and critical thinking. However, limited research exists on its integration into audiology and speech‐language pathology courses. This project aimed to determine the benefits of incorporating lived experience engagements into preprofessional audiology and speech‐language pathology training.

**Method:**

Preprofessional students enrolled for a course in adult auditory (re)habilitation and engaged with PWLE during scheduled class sessions. Six PWLE from different countries, each with unique hearing experiences and various hearing technologies, interacted with the students. Students reflected on their experiences using Gibbs' Reflective Cycle; their reports were retrospectively analysed using inductive content analysis.

**Results:**

Sixty‐four preprofessionals across three universities consented to have their reflections analysed. The value of the engagement resulted in three overarching categories: student experience, patient reported experience and professional practice. Subcategories contributing to the student experience included emotions, perceptions and career goals, with the patient experience category including lack of care, passive listening, setting and significant others. The professional practice category included active listening, trust and care decisions.

**Conclusion:**

Incorporating opportunities for PWLE to share their hearing healthcare journey allows preprofessional students the opportunity to be reflective on aspects such as communication, person‐centred care and empathy. Students can utilise these experiences to identify practical tips for enhancing their own clinical skills and highlight core values for their future roles.

## 1 Introduction

Person‐centred care (PCC) has gained widespread recognition in healthcare and is endorsed by the World Health Organization [1] as a fundamental approach to improving patient outcomes. PCC has been defined as the process by which a patient's individual needs, values and preferences are prioritised [2]. However, it is important to note there is no universal definition and application of PCC [3]. More recently, the International Organization for Standardization [4] established standards emphasising PCC principles, aiming to align healthcare practices with patient needs and individual preferences. With growing recognition of the benefits of holistic and compassionate care, PCC has become an integral part of healthcare systems, influencing policy development, educational frameworks and clinical practice globally [5]. While the adoption of PCC has demonstrated improvements in patient satisfaction and clinical outcomes, challenges remain in fully integrating these principles within existing healthcare structures [6].

Audiology and speech‐language therapy are closely related professions dedicated to assessing and treating hearing and communication disorders. In both fields, PCC emphasises tailoring evaluation and intervention to each individual's unique needs, preferences and circumstances [7]. Rather than focusing solely on diagnosis, PCC prioritises understanding the person's lived experiences, goals and daily communication challenges. This holistic approach promotes patient empowerment, active involvement and improved intervention outcomes [8].

The incorporation of PCC into healthcare education is essential for training future professionals [9]. Given the vocational nature of audiology and speech‐language pathology training, students require structured experiences that expose them to the lived experiences of patients to develop a deeper understanding of PCC and support best professional practice. PCC has been recognised as a core component of curricula across various health disciplines, including medicine, physiotherapy and audiology. However, preprofessional students often have limited opportunities to engage meaningfully with PCC principles, particularly from the patient's perspective [10, 11].

Educational curricula increasingly emphasise the role of persons with lived experience (PWLE) as key stakeholders in shaping training and learning experiences [12]. Patient narratives are particularly valuable in reinforcing PCC principles, as they provide a holistic view of the patient journey, focusing not only on clinical procedures but also on emotional and social aspects of healthcare [13]. PWLE, also referred to as ‘Storytellers’ or ‘Experts by Experience’, offer authentic insights that foster empathy, compassion and critical thinking among students [14, 15]. Reflective practice serves as a complementary strategy in this learning process, further supporting the development of these skills [16, 17].

*Patient narratives are particularly valuable in reinforcing PCC principles, as they provide a holistic view of the patient journey*.

Reflective practice promotes self‐awareness and professional development among both students and practitioners [18, 20]. However, meaningful reflection requires structured guidance to ensure that it extends beyond surface‐level observations. Reflective models, such as Gibbs' Reflective Cycle [20], provide a systematic framework for analysing experiences by addressing emotions, evaluations and action plans for future clinical encounters [21]. Research showcases the utility of Gibbs' model in particular for nursing students to demonstrate and develop empathy and communication skills [22]. By integrating reflective practice with lived experience narratives, students can deepen their understanding of PCC and improve their clinical skills. This integration allows them to critically examine the patient journey, identify areas for improvement and apply these insights into future professional practices [24, 26].

Given the diverse ways in which PCC is conceptualised and implemented across healthcare systems, an international perspective provides valuable insights into its global integration within clinical practice. This study explored how students from multiple countries engage with lived experience narratives and how these experiences shape their understanding of PCC. The study, therefore, aimed to determine the benefits of incorporating lived experience engagements into preprofessional audiology and speech‐language pathology training.

## 2 Method

As part of the learning experience, PWLE of hearing loss were recruited and interviewed by the researchers at their respective universities. Students engaged in the interviews and completed a reflection report on their experience. A retrospective inductive content analysis of the reflection reports was conducted to explore the students experiences. Ethical clearance was obtained from each of the participating institutions' review boards (HUM003/0423, HLS21185, 2410569966).

Furthermore, in order to gain a diverse perspective on the project, a PWLE (Author) was recruited from the IDA Institute's Person‐Centered Hearing Network (https://idainstitute.com/), following their expression of interest in the project. Author (C.H.) was a core member of the research team and participated in all project planning meetings and contributed meaningfully throughout the design and development phases. The input provided streamlined the recruitment process and improved the clarity and accessibility of the preparation materials provided to participants. Author (C.H.) also reviewed and provided feedback on the preliminary results to ensure they aligned with lived experience perspectives. The collaboration strengthened the overall design and implementation, which enriched the study's relevance, rigour and sensitivity to real‐world concerns.

### 2.1 Student Participants

Purposeful sampling was used to recruit participants for this project. The project involved pre‐professional students from universities across three continents, South Africa, United Kingdom and the United States. It examined the global relevance and potential benefits of engaging with a PWLE of hearing loss while taking a course on adult auditory (re)habilitation. The responses included 31 students majoring in communication sciences and disorders or speech‐language pathology from one university and 33 second‐year audiology students from the other two universities. Students provided informed consent for their anonymised reflection reports to be analysed retrospectively for this study.

### 2.2 Courses on Auditory Rehabilitation

Students participating in this project were completing a compulsory module related to adult auditory (re)habilitation at their respective universities from June 2023 to March 2024. The modules were delivered to 138 undergraduate students, of whom 64 students provided informed consent for their optional reflection reports to be analysed retrospectively for this project. The project could be conducted across these three universities as the content being taught in the modules was aligned. The content covered included auditory rehabilitation, hearing aid technology and fitting, PCC approaches, counselling skills and communication strategies for supporting individuals with hearing loss and their families. The modules were presented by the qualified researcher and academic involved in the module and the project. The module content was provided through face‐to‐face classroom sessions, coupled with the use of uploaded PowerPoint slide decks, resources and assignments onto each university's online learning platform (Blackboard).

### 2.3 Procedures

PWLE were recruited through the research team's network, primarily drawing on individuals known through their university‐affiliated clinics. The PWLE were contacted to participate via email or telephone and were not reimbursed for their time and involvement. The goal was to recruit one PWLE per country to allow each group of students to engage with a PWLE from their respective region. PWLE interested in the project received information about the pre‐professional students' learning objectives and the types of information they would be expected to share. Those expressing interest were screened by the researchers (Data S1) to ensure that they were fluent in English (medium of teaching), fitted with hearing aids and/or at least one cochlear implant by an audiologist and had previously engaged with students to align with the focus of the learning activity. Five females and one male PWLE provided consent and were recruited for the project; three had previously participated in educational activities at the respective institutions, one had no previous experience with students, while the other two had teaching backgrounds, specifically in the school educational sector. Five of the PWLE were white and one was Indian. Upon providing informed consent, the PWLE were given a preparation document (Data S2) to help them reflect on their experiences with an audiologist and/or speech‐language pathologist, particularly focusing on PCC principles relevant to auditory (re)habilitation. An orientation session was conducted with the PWLE by the researcher prior to the session with students. This was conducted to familiarise the PWLE with the questions and clarify if and where any information was needed. During the orientation, the PWLE were also encouraged to share both positive and negative examples related to the tenets of PCC, including information sharing, empathy, participation and collaboration.

Prior to the engagement with the PWLE, students were guided on the use of Gibb's [20] Reflective Cycle to support their structured reflection on the experience. Gibbs' Reflective Cycle is commonly used in healthcare education to promote critical thinking, self‐awareness and ongoing professional development among students. The model provides a structured six‐step process that includes description, feelings, evaluation, analysis, conclusion and an action plan. This approach encourages learners to reflect on clinical experiences in a thoughtful and organised way and has been used in other healthcare disciplines [25]. Students in these modules were already familiar with the concept of reflection, having been introduced to various reflective models during earlier academic or practical experiences. However, Gibbs' model was specifically chosen in this context as it provided a clear and structured approach through each stage of the reflective process. By breaking reflection down into six manageable parts, it supports students to develop a deeper understanding of their learning and professional development.

The class engagement session took place during scheduled class time across the three institutions. The 60‐min discussion facilitated by the respective researcher at each university was based on the lived experience of engaging and interacting with an audiologist and/or speech‐language pathologist during the intervention process (hearing evaluation, fitting and support with hearing device/aid, auditory rehabilitation) (Data S3). The learning opportunity allowed the pre‐graduate students to learn firsthand what patients need, providing them with a better understanding of PCC.

### 2.4 Data Collection

As part of the learning opportunity, students were required to reflect on their experience with the PWLE utilising Gibb's [20] Reflective Cycle. The reflection report was optional for all students registered for and completing the academic course at their various universities. Students were required to complete their reflection reports independently and were provided with time to do so in class immediately after the learning opportunity or within 48 hours of the learning opportunity. The reflection report was limited to between 500 and 750 words and was submitted on their online learning platform. Students were required to provide informed consent to anonymise their reflection reports in order to retrospectively analyse their responses to gain an understanding of how the learning opportunity was perceived by them via an electronic link. Of the 138 participating students, 64 students provided informed consent for their reflection reports to be analysed retrospectively for this project.

### 2.5 Data Analysis

The reflection reports were analysed using inductive content analysis [26]. Reflection reports were consolidated on a Microsoft Excel spreadsheet and allocated a numerical participant identity to form a de‐identified dataset. All data were retrospective in nature. The data were first examined through data immersion by four coders (authors). A coder was assigned as lead coder to an average of 16 unique responses and as secondary coder to an average of 16 additional unique responses. Each response was reviewed by a minimum of two coders. An inductive coding process was used where meaningful units were developed by the coder. The lead and secondary coder discussed the meaningful units and developed categories. When discrepancies arose between the two coders, they were discussed. If agreement could not be reached, a third coder from the research team was included to assist in making a decision. Each category was created through the grouping of related subcategories that reflect the complexities of PCC from the perspectives of pre‐professional students in SLP and audiology. Subcategories were clearly defined by the research team into a codebook as they were developed during the data analysis process. Frequency counts are reported alongside the subcategories in Table 1. Frequency counts refer to the number of codes assigned to the category or subcategory. A higher frequency count did not indicate increased meaningfulness to students, but did indicate the student response counts designated to associated categories and subcategories. Related subcategories were later combined to form categories. Audit trails were maintained by the research team to clearly describe the decision‐making process. Authors consistently met to debrief throughout the data analysis period to clearly define and refine categories and subcategories to mitigate potential biases. Additional interpretive themes were not generated in an effort to best reflect the direct perspectives of the student‐respondents. All student responses were analysed and researchers were in agreement that data saturation was achieved given no new category development over the last 15 responses reviewed.

**TABLE 1:** Categories, subcategories, frequency counts and direct quotes.

Category	Subcategory	Frequency counts	Direct quotes
Student experience	Emotions	69	‘During the discussion I had a mixed feeling of emotions. This experience really touched my heart and made me realize what a big impact small things in life really have on our day‐to‐day activities. The good experience was finding out how much of a difference [we] as Audiologists can make in a person's life without even realizing it.’ (Participant 17) ‘I think in our field we have to learn so much factual information so we can accurately diagnose and treat patients that it can be easy to forget about the emotional side of our field.’ (Participant 39)
	Perceptions	28	‘A powerful reminder to me of the impact us, as clinicians and future audiologists, [can have] on patients' lives.’ (Participant 31) ‘This assignment has changed by perspective and allowed me to look at certain situations differently.’ (Participant 58) ‘The experience was extremely insightful on patient‐centered care when building a rapport between patient and audiologist, as well as giving me a newer and truthful perspective of a real‐life patient's life and how it's not necessarily always a negative experience when patients learn they have hearing loss.’ (Participant 24)
	Career goals	87	‘I hope to never make them feel like my time is more valuable than their peace of mind.’ (Participant 55) ‘This really helped me more deeply understand the importance of listening in my future career.’ (Participant 51) ‘As future health care providers, we may see thousands of clients, but they may only have one speech‐language pathologist or audiologist. While diagnosing and treating clients becomes routine to us, it is not routine to our patients, and we need to remember to be as informative and caring as possible.’ (Participant 34)
Patient‐reported experience	Quality of care	30	‘She felt rushed and got the feeling that the audiologist did not truly care.’ (Participant 1) ‘She told her clinician, and they assumed it was nothing and sent her away without even looking at her ears.’ (Participant 39) ‘They also mentioned that they felt like their doctors were on a time crunch and seemed rushed or short with their care. I think that even though there is a schedule to stick to, an effort should still be made to make these patients feel welcome and comfortable.’ (Participant 42)
	Passive listening	21	‘There were multiple times throughout the interview where the guest speakers said they felt like they were on their own during the scary journey, and felt like nobody was listening to them.’ (Participant 52) ‘I have been disregarded as a patient and all it did for me was lessen my motivation to be seen by a healthcare professional.’ (Participant 61)
	Community engagement	7	‘He expressed his hardships whilst trying to shop in public due to the speakers playing loud music above the counter, where he struggled to effectively talk and listen to the clerk.’ (Participant 28) ‘He described how public spaces pretend to cater to those with hearing loss, but do not actually care or put the effort in.’ (Participant 33)
	Family support	26	‘When you have hearing loss, the whole family has to help support and advocate for you and doctors should be more involved with doing the same.’ (Participant 59) ‘Involving family members in a therapy session benefits a patient's mental health.’ (Participant 43) ‘She mentioned that she would've loved her husband to be by her side during the consultation. This once again opened my eyes to the importance of family engagement as an element of person‐centered care.’ (Participant 9)
Professional practice plan	Active listening	78	‘They all mentioned that the providers they like best are the ones who are there to listen to them and treat them like real humans.’ (Participant 42) ‘I learned that active listening is more than just listening to the problem, but includes maintaining eye contact, listening to the patient as a whole, and to what their individual needs are to provide an effective treatment plan.’ (Participant 9)
	Trust	7	‘I realized the importance of actively listening to a patient so that they feel understood, as well as creating a trusting relationship between my client and I.’ (Participant 1) ‘It's very important for the doctor to sit at your level and make eye contact. This builds trust and connection.’ (Participant 59)
	Care decisions	39	‘I learned that almost everything the audiologists did with their clients was carefully noted by the client and it built negative and/or positive stigma about their audiologist … I need to be diligent and attentive in my care if I want to continue to help people.’ (Participant 4) ‘This taught me that I need to slow down throughout my future workdays and treat each patient with the care I would want one of my family members to be treated with as well as the intentionality I would desire from the doctor if I were in the patient seat.’ (Participant 40)

It is important to acknowledge that the authors of this study also served as faculty staff for the participants. All authors had completed training on qualitative research, and two were experienced qualitative researchers. As such, it is necessary to highlight that the authors recognise their own beliefs and experiences may have influenced the analysis and interpretation of the data. Responses were analysed for similarities and differences and assigned to identified categories and subcategories based on similarities.

## 3 Results

**FIGURE 1:**
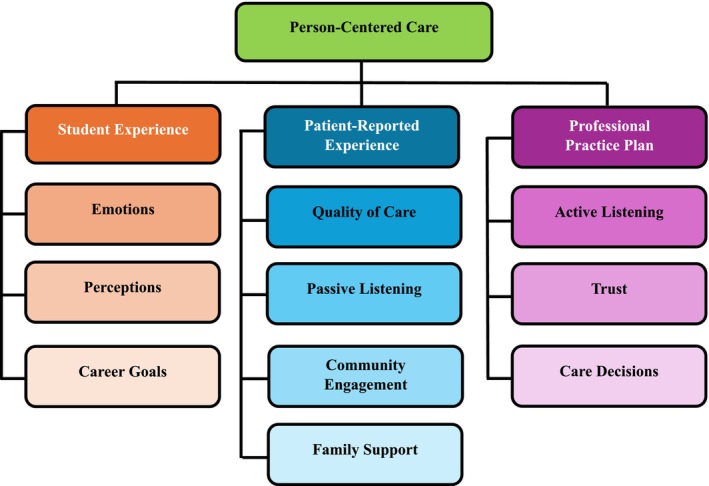
Categories and subcategories.

The reflections of 64 preprofessional students in the field of SLP and audiology across three higher education universities were retrospectively analysed. Given that the retrospective data were gathered from three universities, inconsistent demographic data were available for analysis. Three categories emerged: *student experience*, *patient reported experience* (as perceived by the students) and *professional practice plan* (Figure 1).

### 3.1 Category #1: Student Experience

The category of *Student Experience* (*n* = 184) encapsulates how students perceived their engagement with PWLE, which related to how they felt during the experience and how it expanded their definition of PCC, and how these experiences shaped their career goals. Subcategories of *emotions*, *perceptions* and *career goals* were elucidated from the data to contribute to the main category of student experience.

#### 3.1.1 Emotions

Students expressed a wide range of emotions when reflecting on their exposure to PWLE in the classroom. One student noted,

*I think in our field we have to learn so much factual information so we can accurately diagnose and treat patients that it can be easy to forget about the emotional side of our field*.

Many reported feeling inspired by the potential to make a significant difference in patients' lives, but also expressed sadness and concern with some of the negative experiences reported by the interviewees (Table 1).

#### 3.1.2 Perceptions

For several students, their perceptions of working in their prospective field were altered as a result of their engagement with the PWLE. They reflected on PCC as a holistic approach that impacts all the individuals they serve, including communication partners and caregivers. One student noted,

*The experience was extremely insightful on patient‐centered care when building a rapport between patient and audiologist, as well as giving me a newer and truthful perspective of a real‐life patient's life and how it's not necessarily always a negative experience when patients learn they have hearing loss*.

Students expanded on their PWLE experience to a broader definition of being a healthcare professional (Table 1).

#### 3.1.3 Career Goals

Students identified that they wanted to provide their future patients with resources and information on prevention while also considering variables that would contribute to their patients' overall quality of life. One notable reflection received from a student was,

I hope to never make them [patients] feel like my time is more valuable than their peace of mind.

They also commented on their growth in learning about PCC as well as their desire to learn how they can most effectively provide this care in their future careers (Table 1).

### 3.2 Category #2: Patient‐Reported Experience

The second main category, *patient‐reported experience* (*n* = 84), emerged from students' perceptions of how patients experienced the clinical services they received as well as hearing loss in general. Students often used direct examples from experiences reported by the PWLE to describe the support they felt the patient received. The subcategories of quality of care, passive listening, community engagement and family support emerged as contributors to the development of this category (Table 1).

#### 3.2.1 Quality of Care

Students consistently reflected on comments from the PWLE suggesting that the clinician did not care or disregarded their reported concerns, which was perceived as a negative quality of care by the PWLE and students (Table 1). One student noted,

I think that even though there is a schedule to stick to, an effort should still be made to make these patients feel welcome and comfortable.

Students reflected an appreciation of the importance of accessibility, such as providing clear communication, accommodating diverse needs and the importance of being intentional to make sure individuals feel heard and respected.

#### 3.2.2 Passive Listening

Passive listening, or listening while being disengaged with what is being said, was described by PWLE and cited by students as a contributor to overall patient experience. Several students (*n* = 21) highlighted how this could negatively impact quality of care. In addition to reflecting on their learning experience, students also inherently completed self‐reflections too with one student recalling,

I have been disregarded as a patient and all it did for me was lessen my motivation to be seen by a healthcare professional.

They identified the need to focus on both verbal and non‐verbal communication, remain attentive to the patient's needs, and ask clarifying questions when necessary.

#### 3.2.3 Community Engagement

PWLE described difficulties with activities of daily living such as grocery shopping and engaging in their communities due to poor listening environments. When reflecting on the training, one student recalled how a PWLE reported,

How public spaces pretend to cater to those with hearing loss, but don't actually care or put the effort in.

While this was not directly associated with the quality of care administered by the provider, it was noted by students as a contributor to the overall patient experience.

#### 3.2.4 Family Support

The role of family support and support was reported to be important by the PWLE. One student noted,

Involving family members in a therapy session benefits a patient's mental health. Students recognised the importance of considering and including the family when providing assessment and intervention for an individual with HL. They acknowledged that including family members in care discussions can improve the patient's comfort and willingness to participate in their own care.

### 3.3 Category #3: Professional Practice Plan

The final category, *Professional Practice Plan* (*n* = 124), emerged from students setting goals for how they planned to practice in the future. They reflected on the patient experiences and then identified focus areas for their future practice. Subcategories of *active listening*, *trust* and *care decisions* were elucidated from the data (Table 1).

#### 3.3.1 Active Listening

Students identified active listening as a cornerstone of PCC. Students saw this as a critical component to building trust and a necessary input for making care decisions. One notable reflection from a student was,

They all mentioned that the providers they like best are the ones who are there to listen to them and treat them like real humans.

This experience highlighted the importance of active listening on the part of the clinician.

#### 3.3.2 Trust

Building trust emerged as another component of professional practice. A few students (*n* = 7) discussed how trust develops through transparent communication, care and respect. These students perceived trust to be a foundational necessity for PCC. One student noted,

It's very important for the doctor to sit at your level and make eye contact. This builds trust and connection.

#### 3.3.3 Care Decisions

Students highlighted the importance of involving patients in care decisions, reflecting on the ethical and practical dimensions of PCC. This subcategory was the result of students identifying elements they would consider when they were making care decisions in their future professions while providing PCC (Table 1). One notable student reflection was,

This taught me that I need to slow down throughout my future workdays and treat each patient with the care I would want one of my family members to be treated with as well as the intentionality I would desire from the doctor if I were in the patient seat.

These findings underscore the importance of integrating patient‐centred care (PCC) principles into both the educational training of future clinicians and the practice of current professionals in speech‐language pathology and audiology, aligning with best professional practices to enhance care quality.

## 4 Discussion

This study addressed a gap in the literature pertaining to the student perception of the use of PWLE in application‐based learning. This project aimed to explore how students engaged with narratives from PWLE of hearing loss and how it shaped their understanding of PCC. Through this application‐based reflective learning experience, students developed greater awareness of PCC principles by connecting theoretical content with the real‐world perspectives of PWLE. By undertaking this activity soon after engaging with the PWLE, students were able to undertake both reflection‐in‐action and on‐action [27]. Research on reflective practice in health professionals' education demonstrates the beneficial impacts of introducing reflective practice early in the curriculum that will support future development and opportunities by improving self‐awareness and self‐regulated learning [28]. University education can play a key role in increasing awareness and fostering the development of effective PCC practices among students [29].

*Students developed greater awareness of PCC principles by connecting theoretical content with the real‐world perspectives of PWLE*.

This reflective approach provided students with the opportunity to reflect and set goals for their future clinical practice. These goals included actively listening as care providers, fostering a culture of trust with patients and their families, and intentionally involving patients and their families as active participants in intervention planning. The social–emotional well‐being of adults with hearing loss requires that practitioners take a person‐centred approach to their practice [30]; by incorporating a PWLE assignment into pre‐professional training, students were able to start processing their personal emotions as well as gain insight into patients' lived experiences and emotions that impact their social–emotional well‐being. Initially, many students reported that they had a limited understanding of how hearing loss could impact patients' quality of life. However, as they completed the assignment and reflected on the PCC framework, students began to recognise the importance of individualised care and active listening [1, 9]. Educational experiences like this provide students with an opportunity to challenge or confirm previous assumptions they may have had about working with individuals with hearing loss, thus fostering a more holistic and person‐centred approach in their future professional practice.

Passive listening as a healthcare provider is perceived negatively by patients [31]. Through this engagement, students were able to develop a deeper appreciation for the importance of active listening. Active listening requires intention and is often a skill that must be practiced and refined over time [32]. They recognised that active listening involves not only giving patients space to express themselves but also making intentional efforts to ensure clarity, such as by confirming details, asking follow‐up questions and being mindful of any communication barriers, which aligns with evidence‐based practice [31]. They learned that active listening fosters trust, encourages patients to engage more openly, and ultimately leads to more tailored and effective care. This experience helped students develop a more compassionate, patient‐centred approach to healthcare, where communication is viewed as a two‐way, dynamic process essential to providing best professional practice and the highest quality of care.

Social support, whether it be from the family or community, can impact whether or not an adult seeks intervention for hearing loss as well as if they are satisfied with the intervention they received [36, 38]. Through this experience, students realised that fostering a sense of community requires not only addressing individual communication needs but also creating an environment where patients with hearing loss are encouraged to share their concerns and feel actively involved in their care and decision‐making. They also learned that for many individuals with hearing loss, family members are not only emotional supporters but also vital communication partners, helping to navigate challenges and ensure that healthcare information is fully understood. This collaborative approach enhances the overall healthcare experience, as family members are often key to ensuring continuity of care, advocating for the patient's needs, and fostering a supportive environment at home.

Building onto this, an environment of trust is critical for the success of patients with hearing loss [35]. Students realised that trust is foundational to successful healthcare interactions, especially when patients may already feel marginalised or misunderstood due to communication barriers. Students came to understand that trust is not just about medical expertise but about creating meaningful, collaborative relationships that empower patients to take an active role in their care. Incorporating patients as active participants in their healthcare decisions is well supported in the literature [6, 36, 37], and this study further validates previous findings. This engagement and reflection introduced students to the idea that the provider and patient may not always be on the same page. Students realised that high‐quality care is not solely defined by medical expertise or technical proficiency, but also by the ability of the clinician to foster an inclusive, communicative and empathetic environment. As a result, students may become more attuned to the significance of advocating for accessibility and equity in healthcare settings, recognising that quality care extends beyond assessment and treatment to encompass dignity, inclusion and the promotion of independence.

The importance of the provision of PCC was developed as a goal in students' long‐term career aspirations through this experience. These career goals reflected a deeper commitment to PCC, inclusivity and making a tangible impact on the lives of their patients and their families. Research supports the need for pre‐professionals to start developing reflective practice skills in the fields of audiology and speech‐language pathology [19, 25]. This provided students with a direct opportunity to reflect on working with adults with hearing loss while also identifying opportunities to improve and support best professional practice.

This project is not without limitations; pre‐professional students from different countries, including the United States, South Africa and the United Kingdom, each with distinct healthcare systems, were involved in this study. While the healthcare models varied in terms of patient pathways and financial structures, the PWLE narratives were aligned. This was noted during the analysis of the students' reflections and responses; they were thus grouped together due to the common categories identified, irrespective of the healthcare system. The engagement was also recorded (either live or pre‐recorded); however, to maintain the integrity of the analysis and avoid potential cross‐influence between institutions, the recorded interactions with PWLE were not shared across institutions. Furthermore, the analysis was retrospective and as such the demographic details of the participants were not collected.

## 5 Practical Recommendations for Educators

University education can play a key role in increasing awareness and fostering the development of effective PCC practices among students [29]. Incorporating PWLE into healthcare education is vital to developing compassionate and patient‐focused professionals. To maximise the impact of PWLE involvement, we propose several practical recommendations for clinical educators. PWLE should be involved early and throughout healthcare programmes. Early engagement helps students build empathy and gain insight into patient perspectives, shaping their professional attitudes and behaviours from the beginning. Moreover, incorporating both assessed and non‐assessed reflections can enhance learning in complementary ways. Non‐assessed activities promote honest self‐exploration and personal development, while assessed reflections support the growth of critical thinking and effective communication skills. Furthermore, PWLE should be involved not only as contributors to teaching but also as co‐authors in academic outputs. This reflects a genuine commitment to collaborative practice and reinforces the valuable role of lived experience in shaping both education and professional development, as demonstrated in this research.

*Incorporating PWLE into healthcare education is vital to developing compassionate and patient‐focused professionals*.

To achieve these aims, educators need to offer structured training in reflective skills as a fundamental competency within safe and inclusive learning environments that promote openness and respect. Regular evaluation of PWLE contributions and reflective practice initiatives is essential to share effective approaches and support continuous enhancement. Adopting these strategies will better equip clinical educators to prepare students for the complexities of healthcare, ensuring that patient voices and reflective learning remain central to education and ongoing professional development.

*Educators need to offer structured training in reflective skills as a fundamental competency within safe and inclusive learning environments that promote openness and respect*.

## 6 Future Research

Future research could benefit from including a broader and more representative group of PWLE, particularly from diverse backgrounds across both public and private healthcare sectors in various countries. Engaging PWLE who reflect the local patient population more comprehensively would offer a richer, more nuanced perspective on patient needs and the effectiveness of training. Research emphasises the impact of PWLE in medical education as a means to embed structural changes to curriculum delivery. PWLE provide the insights to living with health conditions that make the theoretical concepts ‘real’ for students [31, 37]. Additionally, seeking feedback directly from PWLE regarding their experiences with students would provide valuable insights into how these interactions can be improved and tailored to better meet both educational and patient care objectives. Finally, exploring the perspectives of teaching and clinical staff within healthcare education would contribute to a deeper understanding of the challenges involved in incorporating PWLE engagements into curricula and help identify strategies for enhancing teaching practices and future educational frameworks.

## 7 Conclusion

By engaging with PWLE of hearing loss, students gained a practical, patient‐centered understanding of care, translating theoretical knowledge into meaningful, real‐world applications. The reflective learning process not only deepened their grasp of PCC principles but also encouraged the development of personal goals for their future clinical practices. The learning opportunity fostered empathy and critical thinking, equipping students with the skills necessary to navigate the complexities of patient care in a diverse and dynamic healthcare landscape, supporting best professional practice.

## Author Contributions

**Rachel Glade:** methodology, formal analysis, writing – original draft, investigation, data curation, visualization, conceptualization. **Saira Hussain:** conceptualization, methodology, data curation, investigation, formal analysis, writing – original draft. **Claire Wilkes:** data curation, investigation, formal analysis, writing – original draft. **Christine Hunter:** methodology, investigation, writing – review and editing. **Faheema Mahomed‐Asmail:** conceptualization, methodology, data curation, investigation, formal analysis, project administration, writing – original draft.

## Ethics Statement

Ethical clearance was obtained from each participating institution's review board (HUM003/0423, HLS21185, 2410569966).

## Consent

All participating students and invited individuals with lived experiences provide informed consent prior to data analysis.

## Conflicts of Interest

The authors declare no conflicts of interest.

## Supporting information


**Data S1:** Supplementary Material.

**Data S2:** Supplementary Material.

**Data S3:** Supplementary Material.

## Data Availability

The data that support the findings of this study are available from the corresponding author upon reasonable request.
