# Supplementary material for: Integrating Lived Experience in Preprofessional Training in Speech Pathology and Audiology
Source: Clin Teach. 2025 Aug 25;22(5):e70183. doi: 10.1111/tct.70183 (PMC12380522; doi:10.1111/tct.70183)
Supplement: Supplementary file 1 — Data S1: Supplementary Material. [file TCT-22-e70183-s001.docx]

**Supplementary digital content 1: Screening Form For Persons with Lived Experience in the Classroom**

**Script:** Thank you so much for contacting us about the opportunity to participate in the Persons with Lived Experience in the Classroom Project. We are looking for individuals with the Lived Experience of Hearing Differences to share their experiences with students in the audiology programs, including their experiences with the hearing healthcare (Audiologist) provider/s.

These experiences are important and can help our students become better clinicians. First, we would like to ask a few questions to get an idea if your experience will fit well within one of our courses. Do you have any questions?

| **Screening Items** | **Answers** |
| --- | --- |
| 1. Name |  |
| 2. What Gender do you identify with? |  |
| 3. What is your age? |  |
| 4. Where are you based? I.e. Rural, urban, semi-urban |  |
| 5.What is your primary language? |  |
| 6. What is your home language? |  |
| 7. When were you diagnosed with a hearing loss? |  |
| 8. Do you use listening technology? When were you fitted and what device/s do you use? |  |
| 9. What prompted you to consider participating in the student engagement activity? |  |
| 6. Can you describe a positive experience you had with your audiologist? |  |
| 7. Can you describe a negative experience you had with your audiologist? |  |
| 8. Do you have experience interacting with students during teaching and learning activities or other forms of engagement? |  |
| 9. Is there anything else you would like to share with me? |  |

Thank you so much for providing this information! If we have an opportunity in one of our classes that is a good fit for you, may we contact you to review the consent forms for this educational student experience? We will also email the consent form to you after we review the steps in the process with you. Do you have any questions?
