# Supplementary material for: Integrating Lived Experience in Preprofessional Training in Speech Pathology and Audiology
Source: Clin Teach. 2025 Aug 25;22(5):e70183. doi: 10.1111/tct.70183 (PMC12380522; doi:10.1111/tct.70183)
Supplement: Supplementary file 2 — Data S2: Supplementary Material. [file TCT-22-e70183-s003.docx]

**Supplementary digital content 2: Preparation document**

For the live interaction, you can prepare examples of the following aspects relating to the IDA person-centered care model:

1. Active listening relates to listening to understand and not fix the problem. This could include how attentive the audiologist was to you, whether they picked up on your subtle cues (discomfort, confusion) and avoided interrupting you.
2. Empathy begins with understanding life from another person’s perspective. This may include the audiologist eye contact, facial expressions, posture, emotion, tone of voice, their ability to listen to you and response to your questions or answers.
3. Open-ended, reflective questions were utilized during the session to engage with you regarding your needs and concerns regarding your hearing ability.
4. The Audiologist displayed understanding of your individual preferences and needs and they were taken into consideration during the consultations.
5. If you had your family and/or friends accompany you to sessions, did the Audiologist include and involve them in the discussion/s.
6. Decisions and goals are made between you and the audiologist to ensure that the goals and rehabilitation options suited your needs.
