# Supplementary material for: Integrating Lived Experience in Preprofessional Training in Speech Pathology and Audiology
Source: Clin Teach. 2025 Aug 25;22(5):e70183. doi: 10.1111/tct.70183 (PMC12380522; doi:10.1111/tct.70183)
Supplement: Supplementary file 3 — Data S3: Supplementary Material. [file TCT-22-e70183-s002.docx]

**Supplementary digital content 3: Facilitators document**

Active listening

1. Empathy
2. Dialogue based on open-ended, reflective questions
3. Understanding of individual preferences and needs
4. Involvement of family and friends
5. Shared goal setting and decision-making

Welcome to todays session. We are so happy that you have volunteered to join us and help us learn more about your journey with a hearing difference. Let’s take a few minutes to for you to introduce us and share some general information about your hearing experience, hearing technology, and your journey. We will keep this part to three minutes. (2-3 min)

Patient journey . . . .

We are very interested in your journey and would like to hear more about your lived experiences. We have shared with you the IDA Institute’s Person Centered Care Model and asked you to think of examples for each of the six principles. We do not want you to mention any specific hospitals, clinics, or providers names - the purpose is for us to learn from you so we can better serve our patients. These examples you choose to share can be positive or negative, it is simply your story as you experienced the care. We are going to provide you with some questions as guides in today’s sessions. Let’s go ahead and get started. We will spend about three minutes on each topic and I will signal to you when you have a minute left.

1. Can you share an experience that you have had with ‘active listening’? What is it about your experience that made you feel like your care provider was or was not ‘actively listening’ to your concerns? (2-3 min)

Response

Thank you for sharing that information - so from this response, we can see how important it is to use nonverbal and verbal behaviors that indicate that you are actively listening to the patient.

*(Or whatever response is appropropriate to highlight the learning point).*

2. We would like to talk about empathy now. Could you share with us a time when you felt like your care provider was successful or not successful in demonstrating empathy during a session? What are some suggestions you might have for future providers? (2-3 min)

Response

Thank you for sharing your experience. This teaches us that empathy is more than putting yourself in your patients shoes. Empathy includes reassuring the patient that your office is a safe place to share their feelings and that regrdless, you acknowledge their feelings about a topic and are there for them to discuss their hearing and balance related issues and will help guide them and provide them with appropriate resources. Even though you cannot possibly experience it from their perspective, you can be there for them and walk through the experience with them. *(Or whatever response is appropropriate to highlight the learning point).*

3. Let’s talk about your experience with providers establishing a relationship with you in such a way that you can have a conversation based on open-ended, reflective questions. What are the specific characteristics that providers have or have not demonstrated when asking open-ended questions about your concerns? Can you identify anything that your hearing care provider has done to make you feel more or less comfortable about shariing? (2-3 min)

Response

Excellent - this example teaches us that active listening and authentic empathy are two of the cornerstones of relationship building that communicates to a patient that this is a safe space to discuss their concerns and to provide you with feedback about their technology. It is not which questions you ask, but how you ask the question and providing enough time for the individual to think about their response. When a patient ‘feels’ rushed, it may cause them to be reticent to ask for the information they need to make decisions. The goal is to understand the patients unique preferences and needs and to apply that information to a solution that fits their concerns. *(Or whatever response is appropropriate to highlight the learning point).*

4. Can you tell us about an experience in which you felt like your care provider embraced your unique preferences and needs or a time in which you felt like the preferences and needs you communicated were disregarded? (2-3 min)

Response

That is a perfect example. In this situation we learned that when a patients who experience recognition of their preferences and needs feel a sense of trust and confidence in their care provider. Sometimes, patients want family members or friends to accompany them to an appointment so they have someone with “good hearing” to also understand the options or recommendations. *(Or whatever response is appropropriate to highlight the learning point).*

5. Can you share with us how your care provider ecourages you to involve family or friends? (2-3 min)

Response

This response teaches us that often the decision to invovle family and friends depends on the context of the situation and that having tools available for captioning the conversation is a good way to involve family and friends when they cannot be present. Apps like InnoCaption can be really helpful for patients who want to share the conversation with others. (Or whatever is appropriate for the response). This brings us to our last principle in patient centered hearing care, and that is the issue of share goal setting and decision-making. *(Or whatever response is appropropriate to highlight the learning point).*

6. Can you tell us about a time when you felt like your were able to participate with your provider in setting goals or in the decision-making process regarding next steps? (2-3 min)

Response

Exactly, so understanding the information and being presented with the pros and cons of each option is really important to you. What we can learn from this example is that it is important to find out about the individual patients unique concerns, needs, and preferences so we can specifically address these in the care plan. *(Or whatever response is appropropriate to highlight the learning point).*

*The instructor (interviewer) wraps up the session by reiterating the 6 person-centered care principles, asking the patient/partner if there is anything else they would like to share with us about their journey, and thanking them again for volunteering to participate in the session.*
